# Supplementary material for: Role and mechanism of NCAPD3 in promoting malignant behaviors in gastric cancer
Source: Front Pharmacol. 2024 Apr 22;15:1341039. doi: 10.3389/fphar.2024.1341039 (PMC11070777; doi:10.3389/fphar.2024.1341039)
Supplement: Supplementary file 11 [file DataSheet2.ZIP › GSEA/Oncogenic signature/my_analysis.Gsea.1599461962066/gsea_report_for_NCAPD3_MUT_1599461962066.html]

Report for NCAPD3\_MUT 1599461962066 [GSEA]

| GS  follow link to MSigDB | GS DETAILS | SIZE | ES | NES | NOM p-val | FDR q-val | FWER p-val | RANK AT MAX | LEADING EDGE || 1 | ALK\_DN.V1\_UP | Details ... | 20 | -0.60 | -2.67 | 0.000 | 0.000 | 0.000 | 316 | tags=70%, list=22%, signal=89% |
| 2 | RB\_DN.V1\_DN | Details ... | 22 | -0.54 | -2.55 | 0.000 | 0.000 | 0.000 | 536 | tags=91%, list=38%, signal=144% |
| 3 | BMI1\_DN\_MEL18\_DN.V1\_UP | Details ... | 28 | -0.47 | -2.44 | 0.000 | 0.003 | 0.004 | 460 | tags=79%, list=33%, signal=114% |
| 4 | P53\_DN.V1\_UP | Details ... | 32 | -0.44 | -2.35 | 0.000 | 0.004 | 0.007 | 498 | tags=78%, list=35%, signal=118% |
| 5 | MEK\_UP.V1\_UP | Details ... | 32 | -0.42 | -2.27 | 0.003 | 0.008 | 0.018 | 573 | tags=81%, list=41%, signal=134% |
| 6 | RAF\_UP.V1\_UP | Details ... | 23 | -0.47 | -2.23 | 0.003 | 0.009 | 0.025 | 401 | tags=65%, list=28%, signal=90% |
| 7 | EGFR\_UP.V1\_UP | Details ... | 33 | -0.40 | -2.21 | 0.002 | 0.009 | 0.028 | 573 | tags=79%, list=41%, signal=130% |
| 8 | KRAS.600\_UP.V1\_UP | Details ... | 17 | -0.52 | -2.16 | 0.000 | 0.011 | 0.040 | 312 | tags=71%, list=22%, signal=90% |
| 9 | ERBB2\_UP.V1\_UP | Details ... | 29 | -0.39 | -2.08 | 0.002 | 0.019 | 0.080 | 573 | tags=79%, list=41%, signal=131% |
| 10 | STK33\_NOMO\_UP | Details ... | 47 | -0.33 | -2.04 | 0.000 | 0.022 | 0.100 | 397 | tags=57%, list=28%, signal=77% |
| 11 | MEL18\_DN.V1\_UP | Details ... | 23 | -0.42 | -2.04 | 0.005 | 0.020 | 0.100 | 460 | tags=74%, list=33%, signal=108% |
| 12 | KRAS.LUNG.BREAST\_UP.V1\_UP | Details ... | 16 | -0.47 | -2.02 | 0.002 | 0.022 | 0.119 | 315 | tags=63%, list=22%, signal=80% |
| 13 | RAF\_UP.V1\_DN | Details ... | 21 | -0.43 | -1.96 | 0.007 | 0.029 | 0.164 | 376 | tags=67%, list=27%, signal=90% |
| 14 | STK33\_UP | Details ... | 47 | -0.31 | -1.95 | 0.000 | 0.029 | 0.173 | 421 | tags=60%, list=30%, signal=82% |
| 15 | ATF2\_UP.V1\_DN | Details ... | 17 | -0.45 | -1.93 | 0.000 | 0.031 | 0.203 | 240 | tags=53%, list=17%, signal=63% |
| 16 | ESC\_J1\_UP\_LATE.V1\_UP | Details ... | 20 | -0.42 | -1.93 | 0.007 | 0.031 | 0.211 | 508 | tags=75%, list=36%, signal=116% |
| 17 | RPS14\_DN.V1\_UP | Details ... | 15 | -0.46 | -1.92 | 0.005 | 0.032 | 0.232 | 482 | tags=80%, list=34%, signal=120% |
| 18 | KRAS.600.LUNG.BREAST\_UP.V1\_UP | Details ... | 21 | -0.40 | -1.91 | 0.009 | 0.032 | 0.244 | 262 | tags=52%, list=19%, signal=63% |
| 19 | CYCLIN\_D1\_KE\_.V1\_UP | Details ... | 20 | -0.42 | -1.90 | 0.008 | 0.032 | 0.253 | 292 | tags=50%, list=21%, signal=62% |
| 20 | LTE2\_UP.V1\_UP | Details ... | 22 | -0.40 | -1.88 | 0.005 | 0.034 | 0.279 | 227 | tags=41%, list=16%, signal=48% |
| 21 | RELA\_DN.V1\_UP |  | 16 | -0.44 | -1.85 | 0.022 | 0.037 | 0.317 | 508 | tags=75%, list=36%, signal=116% |
| 22 | E2F1\_UP.V1\_DN |  | 28 | -0.36 | -1.84 | 0.017 | 0.040 | 0.346 | 418 | tags=64%, list=30%, signal=90% |
| 23 | PRC2\_EZH2\_UP.V1\_UP |  | 21 | -0.39 | -1.81 | 0.009 | 0.044 | 0.391 | 554 | tags=76%, list=39%, signal=124% |
| 24 | LEF1\_UP.V1\_UP |  | 17 | -0.42 | -1.80 | 0.024 | 0.045 | 0.410 | 478 | tags=76%, list=34%, signal=114% |
| 25 | LEF1\_UP.V1\_DN |  | 21 | -0.38 | -1.79 | 0.018 | 0.048 | 0.433 | 132 | tags=38%, list=9%, signal=41% |
| 26 | MTOR\_UP.N4.V1\_UP |  | 37 | -0.32 | -1.78 | 0.010 | 0.047 | 0.440 | 632 | tags=78%, list=45%, signal=138% |
| 27 | PRC2\_EED\_UP.V1\_UP |  | 22 | -0.36 | -1.71 | 0.016 | 0.065 | 0.565 | 410 | tags=64%, list=29%, signal=88% |
| 28 | PTEN\_DN.V2\_UP |  | 15 | -0.41 | -1.69 | 0.028 | 0.070 | 0.607 | 475 | tags=80%, list=34%, signal=119% |
| 29 | LTE2\_UP.V1\_DN |  | 31 | -0.30 | -1.66 | 0.032 | 0.078 | 0.659 | 477 | tags=58%, list=34%, signal=86% |
| 30 | NFE2L2.V2 |  | 44 | -0.27 | -1.65 | 0.025 | 0.080 | 0.679 | 290 | tags=39%, list=21%, signal=47% |
| 31 | AKT\_UP.V1\_UP |  | 23 | -0.34 | -1.62 | 0.030 | 0.094 | 0.756 | 669 | tags=83%, list=47%, signal=155% |
| 32 | PRC2\_SUZ12\_UP.V1\_UP |  | 16 | -0.38 | -1.57 | 0.055 | 0.113 | 0.834 | 244 | tags=44%, list=17%, signal=52% |
| 33 | STK33\_SKM\_UP |  | 36 | -0.27 | -1.57 | 0.051 | 0.111 | 0.836 | 403 | tags=56%, list=29%, signal=76% |
| 34 | ATF2\_S\_UP.V1\_DN |  | 19 | -0.35 | -1.56 | 0.049 | 0.115 | 0.854 | 166 | tags=37%, list=12%, signal=41% |
| 35 | GCNP\_SHH\_UP\_EARLY.V1\_UP |  | 21 | -0.33 | -1.53 | 0.046 | 0.130 | 0.897 | 243 | tags=43%, list=17%, signal=51% |
| 36 | HOXA9\_DN.V1\_DN |  | 18 | -0.34 | -1.51 | 0.063 | 0.140 | 0.915 | 943 | tags=100%, list=67%, signal=298% |
| 37 | CSR\_LATE\_UP.V1\_UP |  | 18 | -0.34 | -1.50 | 0.075 | 0.143 | 0.923 | 658 | tags=83%, list=47%, signal=154% |
| 38 | STK33\_SKM\_DN |  | 27 | -0.29 | -1.46 | 0.095 | 0.167 | 0.950 | 415 | tags=59%, list=29%, signal=82% |
| 39 | IL21\_UP.V1\_UP |  | 17 | -0.34 | -1.44 | 0.073 | 0.173 | 0.956 | 378 | tags=53%, list=27%, signal=71% |
| 40 | GCNP\_SHH\_UP\_LATE.V1\_UP |  | 17 | -0.33 | -1.44 | 0.096 | 0.172 | 0.958 | 311 | tags=41%, list=22%, signal=52% |
| 41 | VEGF\_A\_UP.V1\_UP |  | 20 | -0.31 | -1.44 | 0.083 | 0.168 | 0.958 | 315 | tags=45%, list=22%, signal=57% |
| 42 | ATF2\_S\_UP.V1\_UP |  | 18 | -0.31 | -1.39 | 0.108 | 0.202 | 0.987 | 328 | tags=44%, list=23%, signal=57% |
| 43 | BMI1\_DN.V1\_UP |  | 20 | -0.30 | -1.36 | 0.107 | 0.227 | 0.994 | 476 | tags=65%, list=34%, signal=97% |
| 44 | JAK2\_DN.V1\_DN |  | 16 | -0.31 | -1.29 | 0.192 | 0.294 | 0.999 | 367 | tags=50%, list=26%, signal=67% |
| 45 | HOXA9\_DN.V1\_UP |  | 17 | -0.29 | -1.29 | 0.161 | 0.288 | 0.999 | 250 | tags=47%, list=18%, signal=57% |
| 46 | IL15\_UP.V1\_UP |  | 23 | -0.27 | -1.29 | 0.180 | 0.283 | 0.999 | 527 | tags=65%, list=37%, signal=102% |
| 47 | AKT\_UP\_MTOR\_DN.V1\_UP |  | 20 | -0.27 | -1.24 | 0.198 | 0.336 | 1.000 | 324 | tags=45%, list=23%, signal=58% |
| 48 | RB\_P130\_DN.V1\_DN |  | 15 | -0.29 | -1.22 | 0.223 | 0.349 | 1.000 | 620 | tags=80%, list=44%, signal=141% |
| 49 | IL2\_UP.V1\_DN |  | 15 | -0.30 | -1.22 | 0.211 | 0.343 | 1.000 | 242 | tags=40%, list=17%, signal=48% |
| 50 | MYC\_UP.V1\_DN |  | 16 | -0.28 | -1.18 | 0.241 | 0.389 | 1.000 | 98 | tags=25%, list=7%, signal=27% |
| 51 | E2F1\_UP.V1\_UP |  | 21 | -0.24 | -1.13 | 0.284 | 0.444 | 1.000 | 703 | tags=76%, list=50%, signal=150% |
| 52 | KRAS.DF.V1\_DN |  | 24 | -0.23 | -1.12 | 0.306 | 0.457 | 1.000 | 440 | tags=54%, list=31%, signal=77% |
| 53 | CAMP\_UP.V1\_UP |  | 23 | -0.23 | -1.11 | 0.311 | 0.458 | 1.000 | 1097 | tags=100%, list=78%, signal=442% |
| 54 | TGFB\_UP.V1\_UP |  | 28 | -0.22 | -1.11 | 0.320 | 0.453 | 1.000 | 525 | tags=64%, list=37%, signal=100% |
| 55 | EGFR\_UP.V1\_DN |  | 29 | -0.21 | -1.08 | 0.333 | 0.487 | 1.000 | 314 | tags=34%, list=22%, signal=43% |
| 56 | RAPA\_EARLY\_UP.V1\_UP |  | 16 | -0.26 | -1.07 | 0.378 | 0.490 | 1.000 | 143 | tags=25%, list=10%, signal=28% |
| 57 | ESC\_V6.5\_UP\_LATE.V1\_UP |  | 19 | -0.23 | -1.02 | 0.404 | 0.558 | 1.000 | 825 | tags=84%, list=58%, signal=200% |
| 58 | MTOR\_UP.V1\_DN |  | 18 | -0.23 | -1.02 | 0.432 | 0.552 | 1.000 | 484 | tags=61%, list=34%, signal=92% |
| 59 | RB\_P107\_DN.V1\_DN |  | 17 | -0.23 | -1.02 | 0.410 | 0.543 | 1.000 | 498 | tags=65%, list=35%, signal=99% |
| 60 | CAMP\_UP.V1\_DN |  | 34 | -0.18 | -0.98 | 0.474 | 0.595 | 1.000 | 613 | tags=65%, list=43%, signal=112% |
| 61 | STK33\_NOMO\_DN |  | 38 | -0.17 | -0.98 | 0.476 | 0.587 | 1.000 | 389 | tags=42%, list=28%, signal=57% |
| 62 | MTOR\_UP.V1\_UP |  | 21 | -0.21 | -0.96 | 0.466 | 0.609 | 1.000 | 857 | tags=86%, list=61%, signal=215% |
| 63 | TBK1.DF\_UP |  | 33 | -0.17 | -0.93 | 0.521 | 0.659 | 1.000 | 543 | tags=55%, list=38%, signal=87% |
| 64 | PDGF\_ERK\_DN.V1\_DN |  | 21 | -0.20 | -0.92 | 0.571 | 0.667 | 1.000 | 189 | tags=24%, list=13%, signal=27% |
| 65 | PDGF\_UP.V1\_UP |  | 15 | -0.22 | -0.91 | 0.553 | 0.664 | 1.000 | 279 | tags=33%, list=20%, signal=41% |
| 66 | CSR\_EARLY\_UP.V1\_UP |  | 19 | -0.20 | -0.91 | 0.543 | 0.658 | 1.000 | 272 | tags=32%, list=19%, signal=39% |
| 67 | KRAS.DF.V1\_UP |  | 23 | -0.17 | -0.86 | 0.643 | 0.735 | 1.000 | 497 | tags=52%, list=35%, signal=79% |
| 68 | MTOR\_UP.N4.V1\_DN |  | 35 | -0.15 | -0.85 | 0.660 | 0.730 | 1.000 | 243 | tags=26%, list=17%, signal=30% |
| 69 | TBK1.DF\_DN |  | 46 | -0.13 | -0.78 | 0.766 | 0.826 | 1.000 | 42 | tags=9%, list=3%, signal=9% |
| 70 | ESC\_V6.5\_UP\_EARLY.V1\_DN |  | 20 | -0.17 | -0.77 | 0.753 | 0.840 | 1.000 | 561 | tags=65%, list=40%, signal=106% |
| 71 | TGFB\_UP.V1\_DN |  | 26 | -0.15 | -0.74 | 0.771 | 0.860 | 1.000 | 276 | tags=31%, list=20%, signal=38% |
| 72 | ESC\_V6.5\_UP\_LATE.V1\_DN |  | 23 | -0.15 | -0.71 | 0.851 | 0.889 | 1.000 | 294 | tags=30%, list=21%, signal=38% |
| 73 | VEGF\_A\_UP.V1\_DN |  | 29 | -0.14 | -0.70 | 0.867 | 0.895 | 1.000 | 114 | tags=14%, list=8%, signal=15% |
| 74 | ESC\_J1\_UP\_EARLY.V1\_UP |  | 16 | -0.17 | -0.70 | 0.816 | 0.884 | 1.000 | 56 | tags=13%, list=4%, signal=13% |
| 75 | IL2\_UP.V1\_UP |  | 25 | -0.13 | -0.65 | 0.909 | 0.919 | 1.000 | 572 | tags=60%, list=41%, signal=99% |
| 76 | ESC\_J1\_UP\_EARLY.V1\_DN |  | 22 | -0.13 | -0.62 | 0.923 | 0.931 | 1.000 | 324 | tags=32%, list=23%, signal=41% |
| 77 | CYCLIN\_D1\_KE\_.V1\_DN |  | 21 | -0.13 | -0.59 | 0.928 | 0.943 | 1.000 | 45 | tags=10%, list=3%, signal=10% |
Table: Gene sets enriched in phenotype **NCAPD3\_MUT (3 samples) **[plain text format]****
